# A computational model for lipid-anchored polysaccharide export by the outer membrane protein GfcD

**DOI:** 10.1016/j.bpj.2024.08.012

**Published:** 2024-08-19

**Authors:** Cecilia Fruet, Mikel Martinez-Goikoetxea, Felipe Merino, Andrei N. Lupas

**Affiliations:** 1 Department of Protein Evolution, Max Planck Institute for Biology Tübingen, Tübingen, Germany

## Abstract

Many bacteria are protected by different types of polysaccharide capsules, structures formed of long repetitive glycan chains that are sometimes free and sometimes anchored to the outer membrane via lipid tails. One type, called group 4 capsule, results from the expression of the *gfcABCDE-etp-etk* operon in *Escherichia coli.* Of the proteins encoded in this operon, GfcE is thought to provide the export pore for free polysaccharide chains, but none of the proteins has been implicated in the export of chains carrying a lipid anchor. For this function, GfcD has been a focus of attention as the only outer membrane β-barrel encoded in the operon. AlphaFold predicts two β-barrel domains in GfcD, a canonical N-terminal one of 12 strands and an unusual C-terminal one of 13 strands, which features a large lateral aperture between strands β1 and β13. This immediately suggests a lateral exit gate for hydrophobic molecules into the membrane, analogous to the one proposed for the lipopolysaccharide export pore LptD. Here, we report an unsteered molecular dynamics study of GfcD embedded in the bacterial outer membrane, with the common polysaccharide anchor, lipid A, inserted in the pore of the C-terminal barrel. Our results show that the lateral aperture does not collapse during simulations and membrane lipids nevertheless do not penetrate the barrel but the lipid chains of the lipid A molecule readily exit into the membrane.

## Graphical abstract

**Figure:**
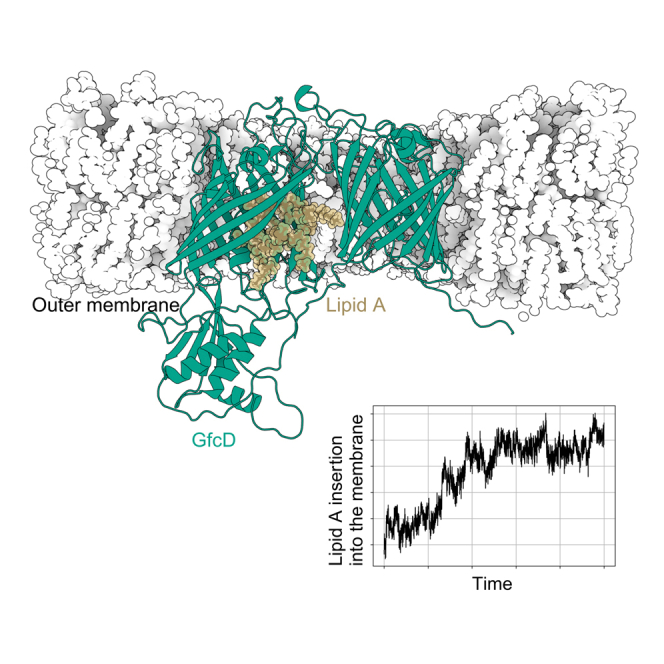

## Significance

Despite the essential role polysaccharide capsules play in the resilience of bacteria to hostile environments, many aspects of their biogenesis are still poorly understood. One aspect concerns the export of capsular polysaccharides carrying a lipid anchor, for which even the proteins mediating the process are unknown. Here, we propose that one of the most widespread families of β-barrel proteins in the bacterial outer membrane is a key agent of this process and show, by biophysical simulation, that it allows the exit of lipid anchors into the membrane through a lateral opening. More generally, our model illuminates the lateral exit mechanisms proposed for the export of hydrophobic macromolecules into the bacterial outer membrane.

## Introduction

The bacterial capsule is a cellular structure that sheathes many bacteria, both gram positive and gram negative (1). It protects cells against changing and often hostile environments, for example against host immunity during infection, and assists in biofilm formation and cell adhesion (2). The capsule is composed of capsular polysaccharides (CPSs), long repetitive chains of several hundred identical oligosaccharide units, differing in their composition and linkage between different capsules. Although their attachment to the bacterial cell surface has not been completely elucidated, it is understood that some CPSs feature a lipid tail that anchors them to the outer leaflet of the outer membrane (3,4,5).

In *Escherichia coli*, there is considerable structural diversity in the CPS oligosaccharide repeat units, but there are only four known biosynthetic pathways for capsule assembly, named groups 1 to 4. The mechanism of capsule biogenesis is only well understood for group 1 (4,5), but some aspects of group 4 can be inferred from the homology of the operons governing the biosynthesis of groups 1 and 4 (4,5). Group 1 capsule production is governed by the *wzabc* operon (3,4,5), where Wza is an octameric translocation channel through the outer membrane, Wzc the corresponding octameric channel through the inner membrane, and Wzb a phosphatase regulating the activity of Wzc. Group 4 capsule production is driven by the *gfcABCDE-etp-etk* operon, of which the last three genes are homologs of *wzabc* (3). The first four genes of the operon, necessary for group 4 capsule expression (6), are homologous to yet another operon of *E. coli*, *yjbEFGH*, which is involved in the secretion of an as-yet uncharacterized exopolysaccharide (3,7,8,9,10). Additionally, the homologs of *gfcBCD* in the gram-negative bacterium *Vibrio anguillarium* are also involved in exopolysaccharide biosynthesis and transport (3,11).

Two of the four proteins encoded in this part of the operon, GfcB and GfcC, are of known structure but unknown activity, while the remaining two, GfcA and GfcD, are entirely uncharacterized at this point. During a recent study, we described a new class of outer membrane β-barrels composed of more than one barrel domain and, surprisingly, came across GfcD and its homologs, which formed the largest cluster of proteins in our dataset (7). These proteins were confidently predicted to contain two barrel domains, an N-terminal one of 12 strands and a C-terminal one, where the assignment of the strand number proved surprisingly difficult, leading us to conclude that it had at least 12 strands but most likely 13 (an unprecedented number for an outer membrane β-barrel).

Our study was published shortly before the AlphaFold protein structure prediction method became available, so one of the first proteins we submitted to the new server was GfcD. As anticipated by us, GfcD was predicted as a multibarrel, with an N-terminal 12-stranded domain and a C-terminal 13-stranded one, connected by a periplasmic middle domain. The structure of the C-terminal barrel caught our attention because it showed a 14-stranded topology missing the last strand, resulting in a lateral aperture between the first (β1) and last (β13) strands. This aperture immediately called to mind other proteins of the outer membrane, for example LptD, PagP, and FadL, which have been proposed to release hydrophobic molecules into the membrane through a lateral gate (12,13,14,15,16,17). For LptD, lipopolysaccharide (LPS) export via a 26-stranded outer membrane β-barrel was simulated by molecular dynamics, showing that although no aperture is visible in the uncomplexed structure, a lateral aperture can open at a weak spot in the barrel, between strands β1 and β26, allowing LPS to exit laterally into the membrane (18).

These observations led us to consider that GfcD might be the pore responsible for the export of lipid-anchored exopolysaccharides in group 4 capsules. To explore the plausibility of this inference, we used molecular dynamics simulations, in analogy to the studies on LptD. Specifically, we simulated the full, mature GfcD protein embedded in an *E. coli* outer membrane patch, with the CPS anchor, lipid A (19), inserted into the C-terminal barrel lumen. In our unsteered molecular dynamics simulations, we consistently observed the tails of lipid A exiting from the lateral aperture in GfcD but no membrane lipids penetrating the pore. These findings support a role for GfcD in the export of lipid-anchored exopolysaccharides and expand the range of outer membrane proteins using the lateral-exit mechanism for the export of hydrophobic molecules.

## Materials and methods

### GfcD modeling and system assembly

No experimental structures of GfcD have been determined, so we employed the model from the AlphaFold2.0 protein structure database (https://www.alphafold.ebi.ac.uk/entry/P75882) (20). To obtain the mature form of the protein, we removed the N-terminal signal sequence (residues 1–18) as detected by SignalP (https://services.healthtech.dtu.dk/services/SignalP-5.0/) (21,22). We then built a complexed version of this mature form by inserting a lipid A moiety interactively into the channel of the C-terminal barrel using Pymol, ensuring that the disaccharide head was oriented toward the extracellular side and that there were no major clashes. Although only some group 4 exopolysaccharides carry a lipid A anchor and the anchor of others is as-yet uncharacterized, we used lipid A for our simulations because it is well established as an anchor in group 4 capsules and because it is the bulkiest anchor known for membrane-anchored surface polysaccharides. We therefore reasoned that if lipid A can exit through GfcD, then other anchors can probably as well. We refined this starting GfcD-lipid A complex with a vacuum energy minimization, keeping the position of all protein heavy atoms restrained. For simulations, we embedded both the complexed and uncomplexed mature GfcD forms into a model *E. coli* outer membrane using CHARMM-GUI (23,24,25,26). This model outer membrane is composed of 1) an outer leaflet of deep rough LPSs, characterized by a minimal core of two 3-deoxy-D-manno-octulosonic acid molecules attached to the lipid A and the absence of O-antigen chains, and 2) an inner leaflet made of a mixture of 1-palmitoyl-2-oleoyl phosphatidylethanolamine, 1-palmitoyl-2-oleoyl-sn-glycero-3-phosphatidylglycerol, and 1,1′-palmitoyl-2,2′-vacenoyl cardiolipin with an 18:1:1 ratio. Such compositions are well established for the simulation of the *E. coli* outer membrane (27,28,29,30). We neutralized the charge on the LPS molecules with Ca2+ ions and further adjusted the ionic strength of the system with 150 mM NaCl. The complex was solvated with TIP3P waters, resulting in systems of 175 × 175 × 145 Å dimension, comprising 483,119 atoms in the uncomplexed version, and 149 × 149 × 170 Å dimension, comprising 347,630 atoms in the complexed version. The assembly procedure is summarized in Fig. S1.

### Molecular dynamics simulations

All simulations were performed in GROMACS 2020.6 (31,32,33) with the CHARMM 36m force field (34,35,36), using the LINCS algorithm to constrain bond vibrations (37). Nonbonded interactions were calculated to a cutoff of 1.2 nm, and van der Waals forces were smoothly decreased to 0 beyond a 1 nm distance. Long-range electrostatic interactions were calculated using particle mesh Ewald (38).

We ran three simulations for the uncomplexed form of GfcD and four independent simulations for the complexed form, one of these without the C-terminal plug. In each case, we performed 30.375 ns of equilibration divided into six consecutive steps, where the system was brought to temperature and atomic restraints were gradually released (summarized in Table S1). In all cases, water molecules rapidly penetrated the channels of the GfcD barrels and filled them completely by step four of the equilibration (Fig. S4). The production runs were 1 *μ*s long, with a 2 fs timestep. The temperature was held at 303.15 K with the stochastic velocity rescaling thermostat (39), using a coupling constant of 1 ps. Pressure was kept at 1 bar using a semi-isotropic Parrinello-Rahman barostat (40,41), using a time constant of 5 ps in all directions and compressibility values of 4.5 × 10^−5^ bar^−1^ for *XY* and *Z*.

### Trajectory analysis

Calculation of angles, root-mean-square deviation (RMSD), and fluctuations were performed with GROMACS. The lipid rotation angle was monitored with a vector connecting the two phosphorus atoms in the lipid A head (PA, PB) in relation to the *Z* axis of the simulation box.

We estimated the insertion of lipid A into the membrane with VMD (42) by computing the fraction of the lipid A area penetrating into the membrane during the simulations.

Other analysis scripts were written in Python using MDAnalysis (43,44).

Molecular graphics have been produced in Pymol, Protein Imager (45), and BioRender (https://www.biorender.com/).

### Sequence analysis and database searches

We performed sequence analyses in the MPI Bioinformatics Toolkit (https://toolkit.tuebingen.mpg.de/) (46,47) using database versions of August 2022. We identified AlphaFold models for GfcD homologs using a BLAST search against the alphafold_uniprot50 database, which gave us access to precomputed AlphaFold models contained in the UniProt database (https://www.uniprot.org/), filtered to a maximum pairwise sequence identity of 50%.

## Results

### The GfcD C-terminal barrel contains a lateral gate into the membrane

**Figure 1:**
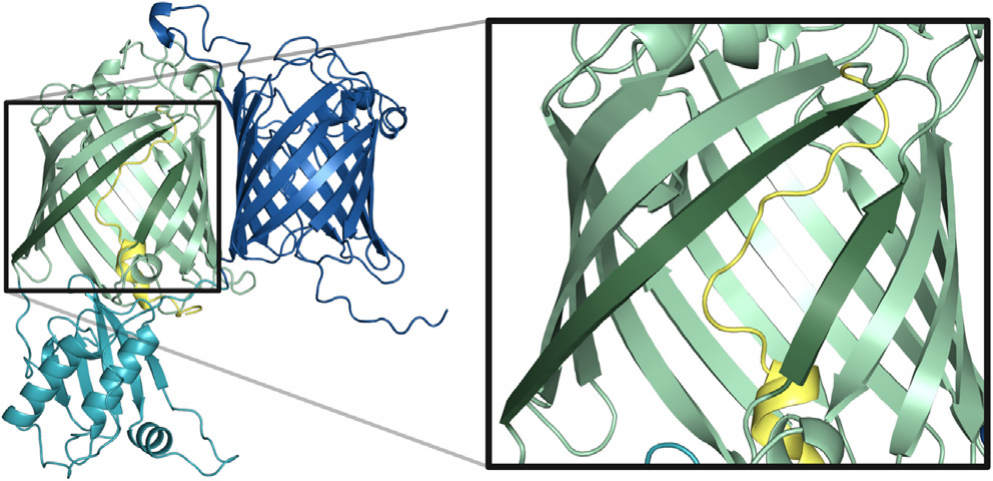
View of GfcD, showing its three domains: GfcD-N (*blue*; residues 19–278), GfcD-M (*cyan*; residues 279–423), and GfcD-C (*green*; residues 424–698), with its C-ter plug highlighted in yellow (residues 674–698). The inset provides a closeup of the lateral gate in GfcD-C.

In the absence of experimental GfcD structures, we used an AlphaFold model for biophysical simulations of the protein. This model is composed of two membrane-embedded β-barrels, separated by a periplasmic domain, in close agreement with a previous computational study based on sequence comparisons and coevolution analysis (7). The N-terminal barrel (GfcD-N) is a canonical 12-stranded structure, but the C-terminal one (GfcD-C) is a highly unusual form, which has the topology of a 14-stranded barrel but is composed only of 13 strands. The lack of the 14th strand in this fold results in a lateral opening between strands β1 and β13, which leads directly into the membrane (Fig. 1). Furthermore, the C-terminal end of the protein is folded into the channel formed by this barrel, in a manner reminiscent of a plug (Fig. 1). Combined, this led us to the hypothesis that the C-terminal barrel is responsible for the export of lipid-anchored exopolysaccharides, with the lateral pore acting as a gate for the lipid anchor to enter the membrane. To test the reliability of the topology we predicted for GfcD, we extracted all AlphaFold2 models of GfcD homologs from the AlphaFold-UniProt database and sampled this dataset at sequence identities to GfcD ranging from 45% to 25% (Fig. S2). Superposition of the mature form of these five models with GfcD yielded pairwise RMSD measurements of between 2.0 and 3.2 Å. Limiting the alignment to the C-terminal barrel yielded even lower pairwise RMSD values between 0.8 and 1.9 Å (Fig. S2). Thus, the overall fold is essentially the same in all models. More importantly, the lateral opening between strands β1 and β13 was clearly apparent and of similar dimensions in all models (Fig. S2).

### The lateral gate in GfcD is stable in molecular dynamics simulations

**Figure 2:**
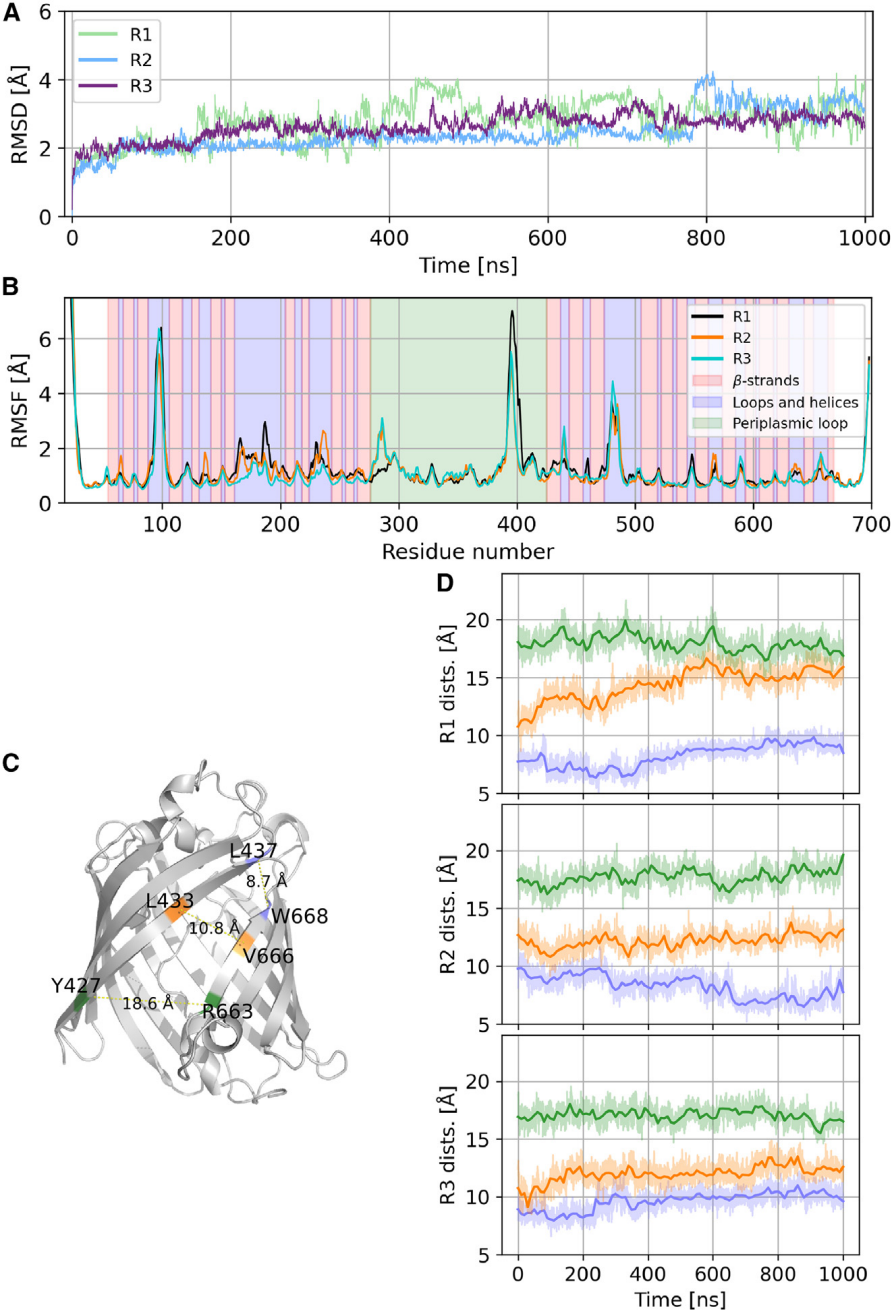
The lateral aperture of GfcD is stable in the uncomplexed form. (*A*) RMSD of the protein backbone. (*B*) RMSF of the protein backbone, with the secondary structure of GfcD highlighted in the background. (*C*) Three reference distances with which we measure the aperture (L437-W668, L433-W666, Y427-R663). (*D*) Time evolution of the three distances shown in (*C*).

To test the stability of the predicted GfcD structure, we inserted the full, mature protein into a model outer membrane and performed three 1-*μ*s-long molecular dynamics simulations (see materials and methods). We observed backbone RMSD values between 2 and 4 Å relative to the starting structure (Fig. 2), consistent with values reported for other membrane-embedded β-barrel protein simulations (48,49,50,51,52,53). Backbone root-mean-square fluctuation (RMSF) was also low except in four distinct regions of high flexibility (RMSF > 4 Å): the regions at the N and C termini, the extracellular loop around residue 230, and the junctions between the periplasmic domain and the two barrels (Fig. 2). Overall, the secondary structure and fold of GfcD was stable throughout the simulations, consistent with previous studies of membrane-embedded β-barrels (48,50). Importantly, in the simulations, the lateral gate remains unchanged. To further explore the behavior of this region of the protein, we monitored the gate size by measuring the distances between the Cα carbons of three pairs of residues, evenly distributed along the gate (LEU437-TRP668, LEU433-VAL666, THR427-ARG663). For all measured distances, the aperture barely fluctuates. Certainly, it does not close (Fig. 2), further supporting the hypothesis that this represents a bona fide lateral exit into the membrane. Interestingly, while the gate remains open, lipids from the membrane never fully enter into the central channel, but rather, their aliphatic tails occasionally flicker across the gate opening, never penetrating farther than about 4 Å (Fig. S3). We attribute this to the fact that the central channel of GfcD-C, despite its relatively hydrophobic characteristics, is fully hydrated (Fig. S4).

### GfcD can accommodate lipid A inside the GfcD-C channel without conformational changes

**Figure 3:**
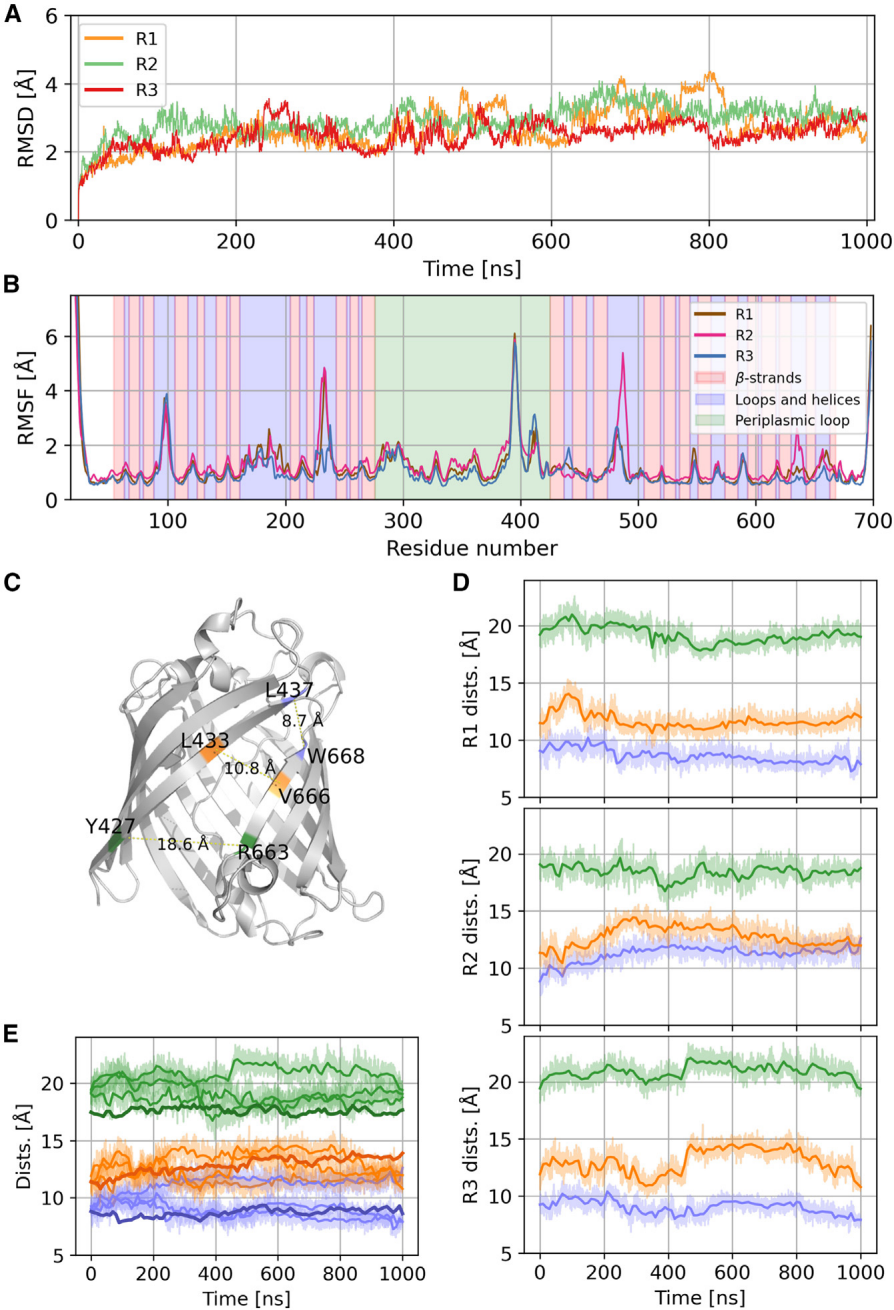
The lateral aperture of GfcD is stable in the simulations with lipid A in the GfcD-C channel. (*A*) RMSD of the protein backbone. (*B*) RMSF of the protein backbone, with the secondary structure of GfcD highlighted in the background. (*C*) Three reference distances with which we measure the aperture (L437-W668, L433-W666, Y427-R663). (*D*) Time evolution of the three distances shown in (*C*). (*E*) Aggregated plots for the time evolutions shown in Fig. 2*D* (mean of the three uncomplexed runs) and Fig. 3*D* (with lipid A). To facilitate the comparison, the average lines for the uncomplexed simulations are shown in bold.

To explore whether the lateral gate can allow the exit of lipid-anchored exopolysaccharides into the outer membrane, we introduced lipid A into the channel of GfcD-C and performed a new set of 1-*μ*s-long molecular dynamics simulations of the full, mature protein. As with the empty GfcD, the protein remained stable during the simulation, with similar backbone RMSD and RMSF values (Fig. 3). Here as well, the lateral pore remained unchanged throughout the simulation (Fig. 3). A comparison with the empty structure (Fig. 3 *E*) shows that the presence of lipid A only results in a slight widening of the periplasmic end of the gate; the rest of the gate remains the same. Further, the consistently low RMSD values across simulation time show that even though GfcD is a multidomain protein, these domains do not substantially move relative to each other whether or not lipid A is present.

Despite its large size, lipid A can be fully accommodated in the GfcD-C barrel even with the C-terminal extension present in the channel, suggesting that if this acts as a plug, it does not obstruct the access of lipid A. To test this, we removed the extension (residues ASP674–GLN698) and ran a further 1-*μ*s-long simulation. As expected, we saw no difference in the behavior of lipid and protein (Figs. S5 and (S6). A hydration layer is always present between lipid A and the protein, highlighting that the molecule can still move with relative freedom.

**Figure 4:**
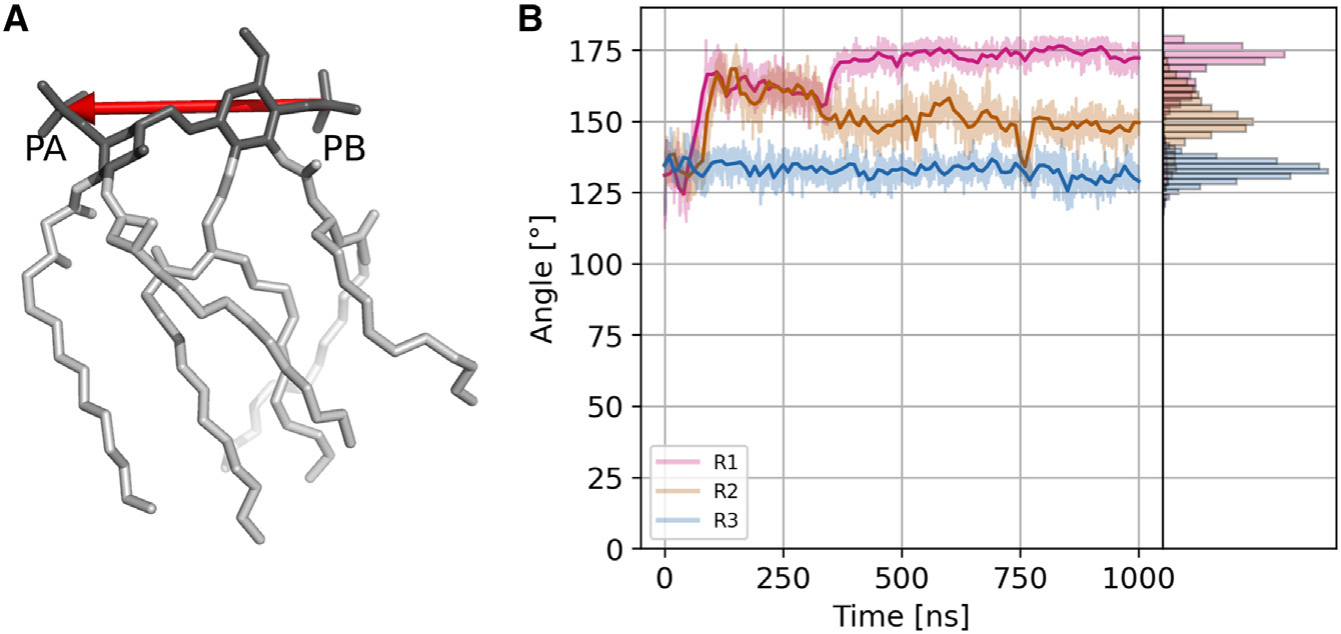
Lipid A spontaneously tilts inside the GfcD-C channel at an angle between 112.5° and 180°. (*A*) The orientation vector used for monitoring the tilting angle of lipid A during the simulations. The lipid A head is shown in dark gray. (*B*) Line plot of the tilting angle of lipid A as a function of time, with histograms on the right.

To further characterize the behavior of lipid A in the protein channel, we monitored the tilting angle of the molecule by tracking the orientation of its headgroup throughout the simulation time, the lipid tails being too flexible to be used as indicators. We considered the tilting angle to be relevant for the export process because the gate in GfcD-C is angled relative to the channel, with an inclination set by the shear number of the β-strands in the barrel. While the orientation adopted by lipid A is different in each independent simulation, with values between 112.5° and 180° (Fig. 4), the orientation becomes relatively stable after 400 ns. This variability between replicas is expected, as lipid A should not have a specific binding site within the channel. Most importantly, though, despite the considerable span of values, all the angles in the set allowed a rotation of the lipid such that its tails exited the gate.

### The aliphatic tails of lipid A spontaneously cross the lateral gate in GfcD-C to enter the membrane

**Figure 5:**
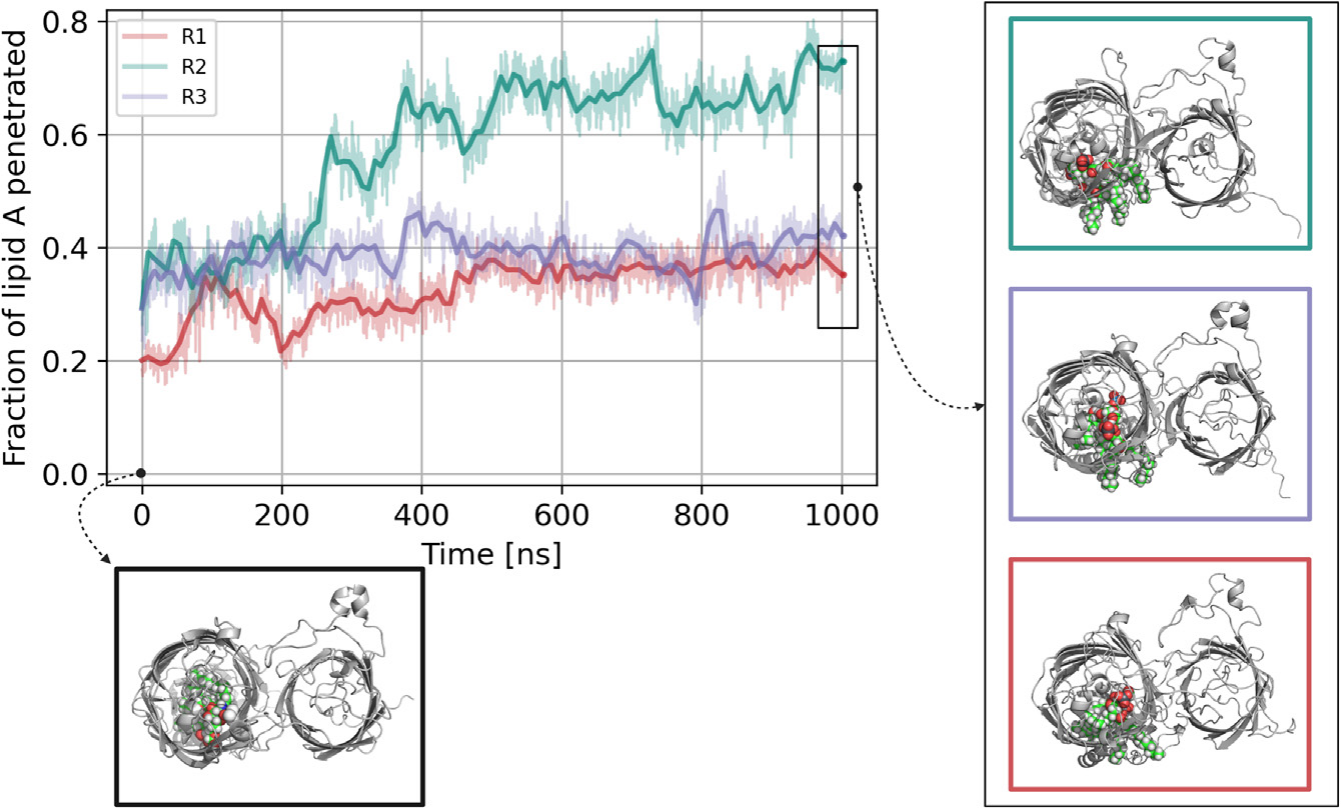
Lipid A exits the GfcD-C channel in all our simulations. The top left image shows the time evolution of the proportion to which the aliphatic tails of lipid A penetrate the membrane. The other four images show snapshots of the initial and final configurations of GfcD, with lipid A highlighted in green to visualize the egress of its lipid tails. The initial snapshot is framed in black, and the three final snapshots are framed in the color of their corresponding time evolution graph.

Although we simulated lipid A without any biasing force, all our simulations show at least partial egress of its lipid tails toward the membrane (Fig. 5; Videos S1, S2, and S3). To quantitatively assess this phenomenon, we measured the extent to which the aliphatic tails penetrated into the membrane throughout the simulations (Fig. 5). In all cases, the lipid A tails rapidly exited the channel to at least 30%, in two replicas within the first few nanoseconds, but in the third, more gradually to 100 ns. This did not correspond to a partial membrane exposure of all six lipid tails but rather to the complete exit of one tail, which was the one closest to the PB phosphate group in all three simulations, followed by the nearest second and third tails. In replica 1 (*red* in Fig. 5), the exit proceeded gradually, and we observed a retreat of the second tail back into the barrel, followed by its almost complete re-emergence over the next 300 ns, after which the process stalled. In replica 3 (*purple* in Fig. 5), the process also stalled after the initial rapid exit of the first two lipid tails, but toward the end of the runtime, a third lipid tail started to exit before the second had fully done so. Only in replica 2 (*cyan* in Fig. 5) did the exit proceed to completion within the runtime, i.e., to the full membrane insertion of all six tails, albeit with occasional partial retreats of tails that had already started to exit.

Video S1. Simulation of lipid A in the channel of GfcD-C, replica 1

Video S2. Simulation of lipid A in the channel of GfcD-C, replica 2

Video S3. Simulation of lipid A in the channel of GfcD-C, replica 3

**Figure 6:**
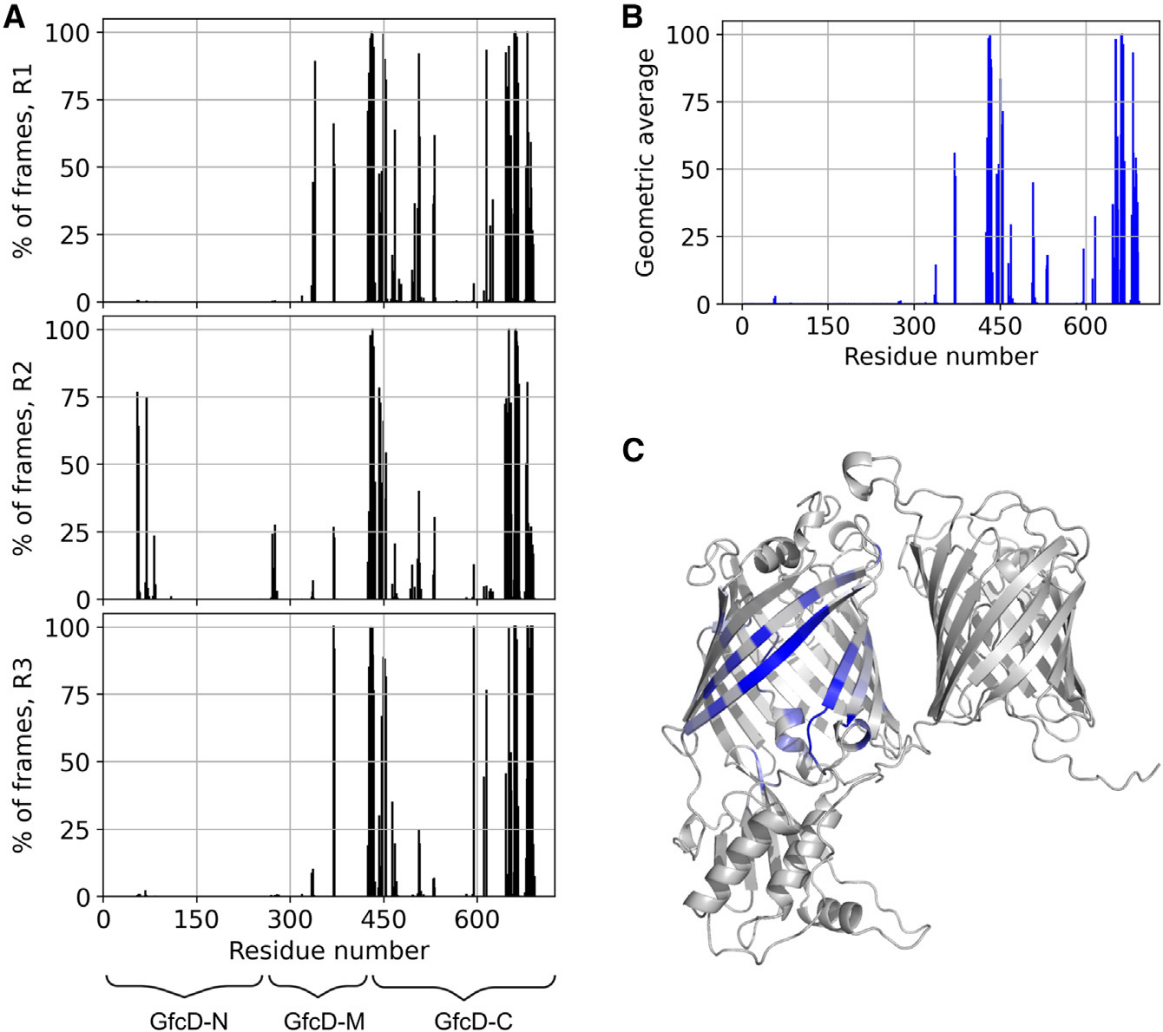
The contacts between GfcD and lipid A characterize the parts of GfcD-C that are directly involved in lipid A export. (*A*) Percentage of frames during which lipid A was in contact with residues in GfcD for each replica. (*B*) The geometric average of the data shown in (*A*). (*C*) The geometric average mapped onto the structure of GfcD.

In order to establish whether any parts of the channel were required to guide lipid A toward the gate in addition to the residues lining the gate itself, we performed a contact-based analysis of the interactions between lipid A and GfcD residues. We monitored these contacts for each trajectory frame of all three replicas and could thus identify specific residues interacting consistently with lipid A (Fig. 6). We calculated a contact score for each residue as the geometric average of the number of its contacts with lipid A in each simulation and mapped the result onto the structure of GfcD (Fig. 6). This analysis established that reproducible contacts with lipid A occur only in strands β1, β2, β12, and β13, which flank the gate, in a loop of the periplasmic domain closest to the GfcD-C channel and in the adjacent part of the C-terminal extension. In the one replica in which the tails of lipid A exited to completion, they even contacted the GfcD-N barrel (Fig. 6). As expected, these contacts were not observed in the two replicas in which the lipid A tails had only exited partially.

## Discussion

In this study, we explored one of the mechanisms by which lipid-anchored CPSs might be exported in bacteria with an outer membrane. We found that the GfcD family of proteins provides an attractive candidate for performing this function. GfcD homologs form the largest group of proteins with more than one barrel domain in the outer membrane of gram-negative bacteria and are present widely in species of this group, tracking the widespread occurrence of exopolysaccharide capsules (7). Previous studies have established that *gfcABCD* homologs are involved in exopolysaccharide biosynthesis and transport (3,7,8,9,10,11). Here, we used unsteered molecular dynamics simulations to study the full-length, mature GfcD protein of *E. coli* in a model outer membrane patch, with and without lipid A as a membrane anchor inserted into the GfcD C-terminal barrel. Our results provide further support for an activity of GfcD in the export of lipid-anchored polysaccharides and outline a mechanism for this process.

In our analyses, the lateral gate in the C-terminal barrel of GfcD (GfcD-C) proved to be consistently predicted across the full range of homologs, from nearly identical ones to ones that had diverged into the “midnight zone” of homology below 20% sequence identity (54). This gate was stably open in molecular dynamics simulations but prevented the penetration of membrane lipids, possibly because of the full hydration of the GfcD-C channel, while allowing the efficient exit of the lipid tails in lipid A.

The middle domain of GfcD, GfcD-M, which is located at the periplasmic entrance of GfcD-C, could be considered a regulator of this exit process. The C-terminal plug of GfcD-C could also be part of a gating mechanism, acting synergistically with GfcD-M to regulate access to the GfcD-C lumen, but we found that this plug does not impede the positioning of the lipid anchor inside the GfcD-C channel and that its removal does not lead to significant changes in the lateral exit of the lipid tails. Its possible role in regulating exopolysaccharide exit thus remains to be elucidated.

We used lipid A in our simulations because it is the most frequent membrane anchor of surface polysaccharides and a proven anchor of group 4 exopolysaccharides, but our model does not exclude other lipid-derived molecules as anchors. Rather, as we observe, lipid A makes no specific contacts to the GfcD-C channel (Fig. 6) and remains surrounded by a shell of water molecules throughout our simulations, showing clearly that it is no ligand of GfcD but a passenger moiety in transit from the cytosol to the outer leaflet of the outer membrane. There is no reason to suspect from our simulations that other lipid anchors would fare any differently in the pore. Indeed, that is what we would expect from an export pore evolved to facilitate the efficient exit of a molecule to its target location. Any specific interactions would slow down the process and would, therefore, be selected against.

Concerning the molecular mechanism underlying the export process, we hypothesize that the saccharide moieties of the exopolysaccharides traverse the channel first, and the entropy gain resulting from their hydration provides a strong pulling force that drags the whole molecule toward the environment. Once the exopolysaccharide has gone through the channel and lipid A is inserted in GfcD-C, it spontaneously tilts, allowing its hydrophobic tails to exit the channel through the lateral gate into the membrane, in a process similar to that envisaged for LPS export through LptD (13,14,18,55). Thus, after the exopolysaccharide chain has fully exited into the extracellular space, the lipid A headgroup follows but remains anchored to the outer leaflet of the outer membrane through its lipid tails, which have exited laterally. In our simulations, the tails do not exit the GfcD-C channel simultaneously but in a sequential process that may stall partway through. We envisage that the pulling force exerted by the hydration of the saccharide moieties could be important to overcome such energy barriers during the export process.

Overall, our study provides a viable hypothesis for the biological role of GfcD homologs in the export of lipid-anchored exopolysaccharides, with potentially broader implications for the lateral-exit model of hydrophobic macromolecule export into the bacterial outer membrane through lateral gates in outer membrane β-barrels. This study provides a functional annotation for one of the most widespread protein families of the bacterial outer membrane.

## Acknowledgments

This work was supported by institutional funds from the 10.13039/501100004189Max Planck Society. The molecular dynamics simulations were performed using the Raven cluster, part of the Max Planck Computing and Data Facility (https://www.mpcdf.mpg.de).

C.F. acknowledges Martin Oettel, Raffaello Potestio, and Thomas Tarenzi for helpful and insightful discussions. C.F. is grateful to the Universities of Trento and Tübingen for the scholarship awarded for participation in the double degree program between the two universities.

## Author contributions

C.F. set up and performed all molecular dynamics simulations, analyzed data, generated the figures, and co-wrote the article; M.M.-G. performed the bioinformatics analyses, advised the data analysis, and co-wrote the article; F.M. advised the molecular dynamics simulations and data analysis and co-wrote the article; and A.N.L. conceptualized the project, guided the bioinformatic analyses, provided the funds for the project, and co-wrote the article. All authors participated in generating the final form of the article.

## Declaration of interests

The authors declare no competing interests.

## Supporting material

Document S1. Figures S1–S6, Tables S1, and S2

Document S2. Article plus supporting material
