# Supplementary material for: A computational model for lipid-anchored polysaccharide export by the outer membrane protein GfcD
Source: Biophys J. 2024 Aug 19;123(20):3491–9. doi: 10.1016/j.bpj.2024.08.012 (PMC11494523; doi:10.1016/j.bpj.2024.08.012)

Biophysical Journal, Volume *123*

Supplemental information

A computational model for lipid-anchored polysaccharide export by the outer membrane protein GfcD

Cecilia Fruet, Mikel Martinez-Goikoetxea, Felipe Merino, and Andrei N. Lupas

Supplemental Material

A computational model for lipid-anchored polysaccharide export by the outer-membrane protein GfcD

Cecilia Fruet[1,2,3], Mikel Martinez-Goikoetxea[1], Felipe Merino[1,4], Andrei N. Lupas[1,*]

1 Department of Protein Evolution, Max Planck Institute for Biology Tübingen, D-72076 Tübingen, Germany

2 Present address: Institute of Bioengineering, School of Life Sciences, École Polytechnique Fédérale de Lausanne (EPFL), CH-1015 Lausanne, Switzerland

3 Present address: SIB Swiss Institute of Bioinformatics, CH-1015 Lausanne, Switzerland

4 Present address: Cube Biotech GmbH, D-40789 Monheim, Germany

***** Corresponding author: andrei.lupas@tuebingen.mpg.de

Figure S1. The procedure we used to set up the simulation box for the unliganded and liganded simulations.

Uncomplexed version

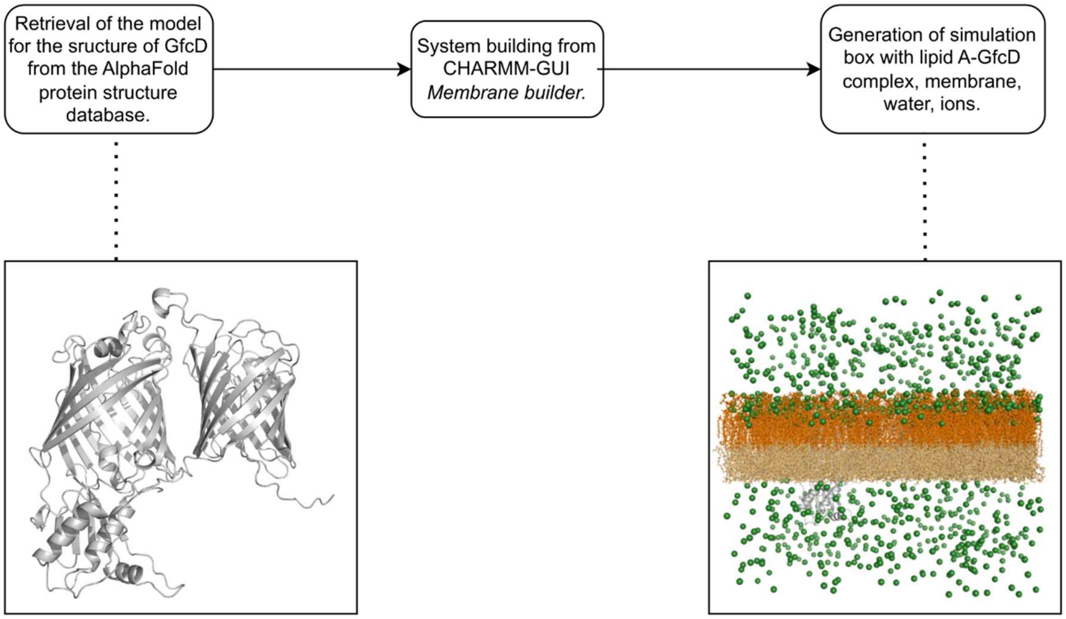

Complexed version

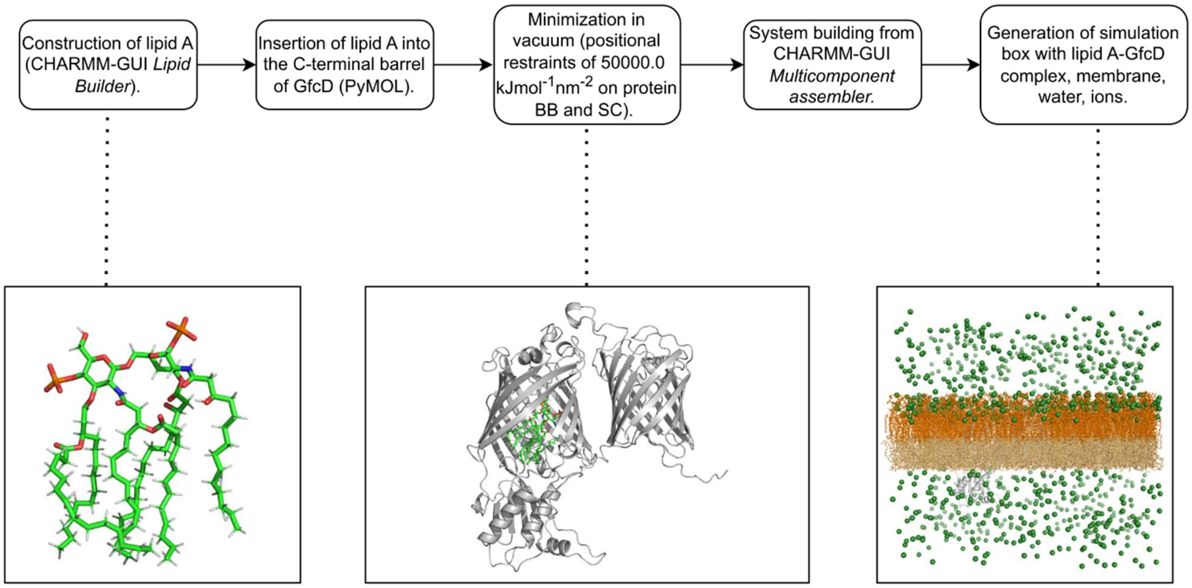

Table S1. Summary of the equilibration protocol for our simulations.

	Equilibration phases					
	NVT	NVT	NPT	NPT	NPT	NPT
Number of steps	1.25×10^5	1.25×10^5	1.25×10^5	5×10^6	5×10^6	5×10^6
Timestep [ps]	1×10^{-3}	1×10^{-3}	1×10^{-3}	2×10^{-3}	2×10^{-3}	2×10^{-3}
Constraint algorithm	LINCS	LINCS	LINCS	LINCS	LINCS	LINCS
Constraints	H-bonds	H-bonds	H-bonds	H-bonds	H-bonds	H-bonds
Thermostat	Berendsen	Berendsen	Berendsen	Berendsen	Berendsen	Berendsen
Reference temperature [K]	303.15	303.15	303.15	303.15	303.15	303.15
Time constant [ps]	1.0	1.0	1.0	1.0	1.0	1.0
Barostat	/	/	Berendsen	Berendsen	Berendsen	Berendsen
Coupling type	/	/	semi-isotropic	semi-isotropic	semi-isotropic	semi-isotropic
Reference pressure [bar] (same in all directions)	/	/	1.0	1.0	1.0	1.0
Compressibility [bar^{-1}] (same in all directions)	/	/	4.5×10^{-5}	4.5×10^{-5}	4.5×10^{-5}	4.5×10^{-5}
Time constant [ps] (same in all directions)	/	/	5.0	5.0	5.0	5.0
Protein backbone restraints [kJ mol^{-1} nm^{-2}]	4000.0	2000.0	1000.0	500.0	200.0	50.0
Protein sidechains restraints [kJ mol^{-1} nm^{-2}]	2000.0	1000.0	500.0	200.0	50.0	0.0
Lipid restraints [kJ mol^{-1} nm^{-2}]	1000.0	400.0	400.0	200.0	40.0	0.0
Dihedral restraints [kJ mol^{-1} nm^{-2}]	1000.0	400.0	200.0	200.0	100.0	0.0

Table S2. Summary of the simulations we ran.

Simulation type	Replicas	Production length
Membrane + GfcD	3	1 μs
Membrane + GfcD + IA	3	1 μs
Membrane + GfcD + IA (no C-ter plug)	1	1 μs

Figure S2. (A) Comparison of GfcD to five homologs, selected to span the range of sequence identities to GfcD from 45% to 25%. The proteins are (1) GfcD, (2) UniProt ID A0A7X4W9T3, (3) A0A2N7DHB9, (4) A0A522EX48, (5) A0A2W5NHM9, and (6) A0A2D7DXB3. The pairwise sequence identity matrix was obtained with Clustal Omega (https://www.ebi.ac.uk/Tools/msa/clustalo/). (B) Pairwise Cα-RMSD (left) and superposition (right) for the C-terminal barrels of the proteins in Panel A.

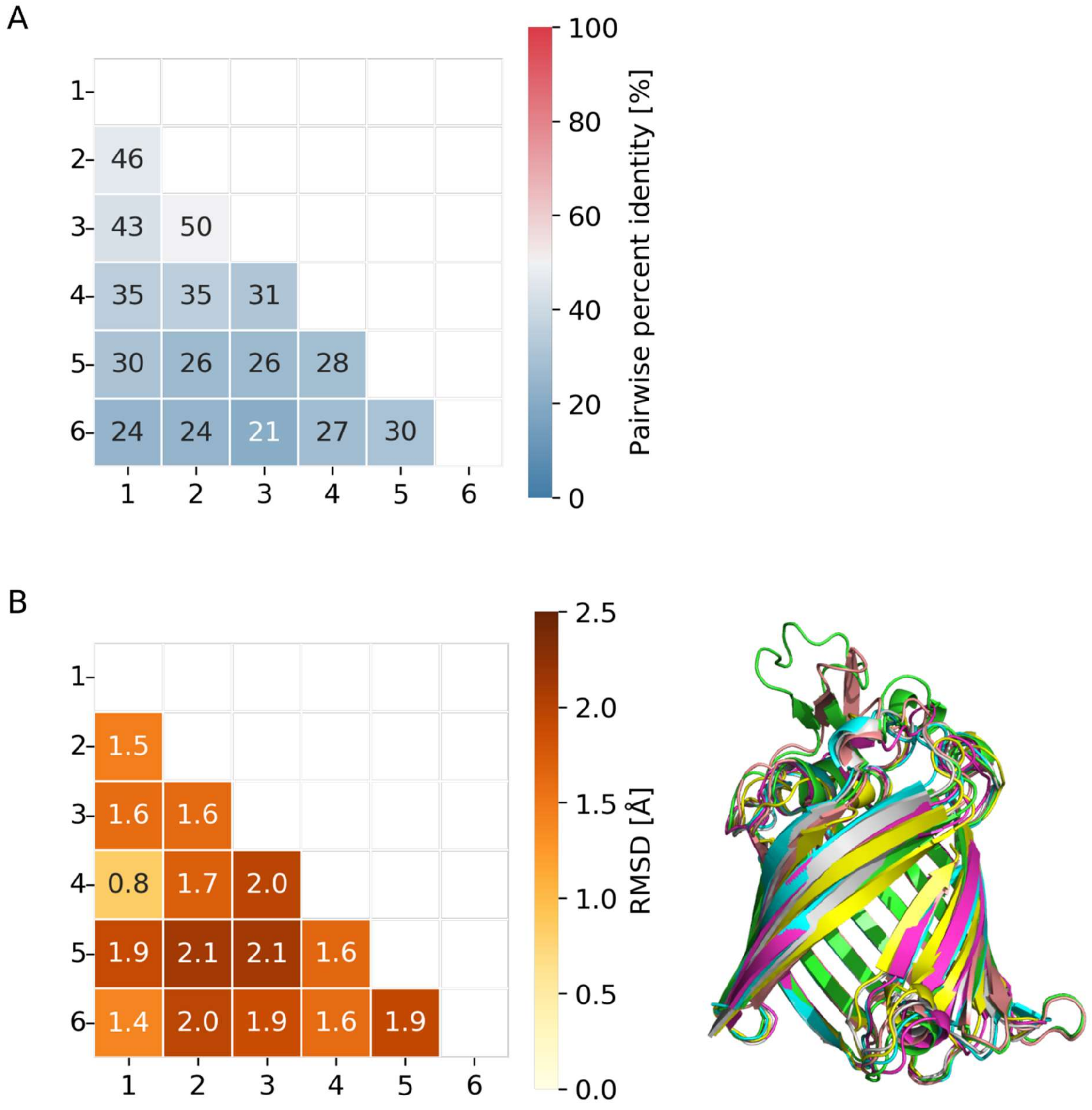

Figure S3. Lipids do not stably enter the channel of GfcD-C in our simulations. Density maps for the the lipids along the trajectories with uncomplexed GfcD (right column), distance of lipids from the gate (left column). The gate position is shown with a red dashed line. Data from replicas 1, 2, and 3 are shown in panels A, B, and C, respectively.

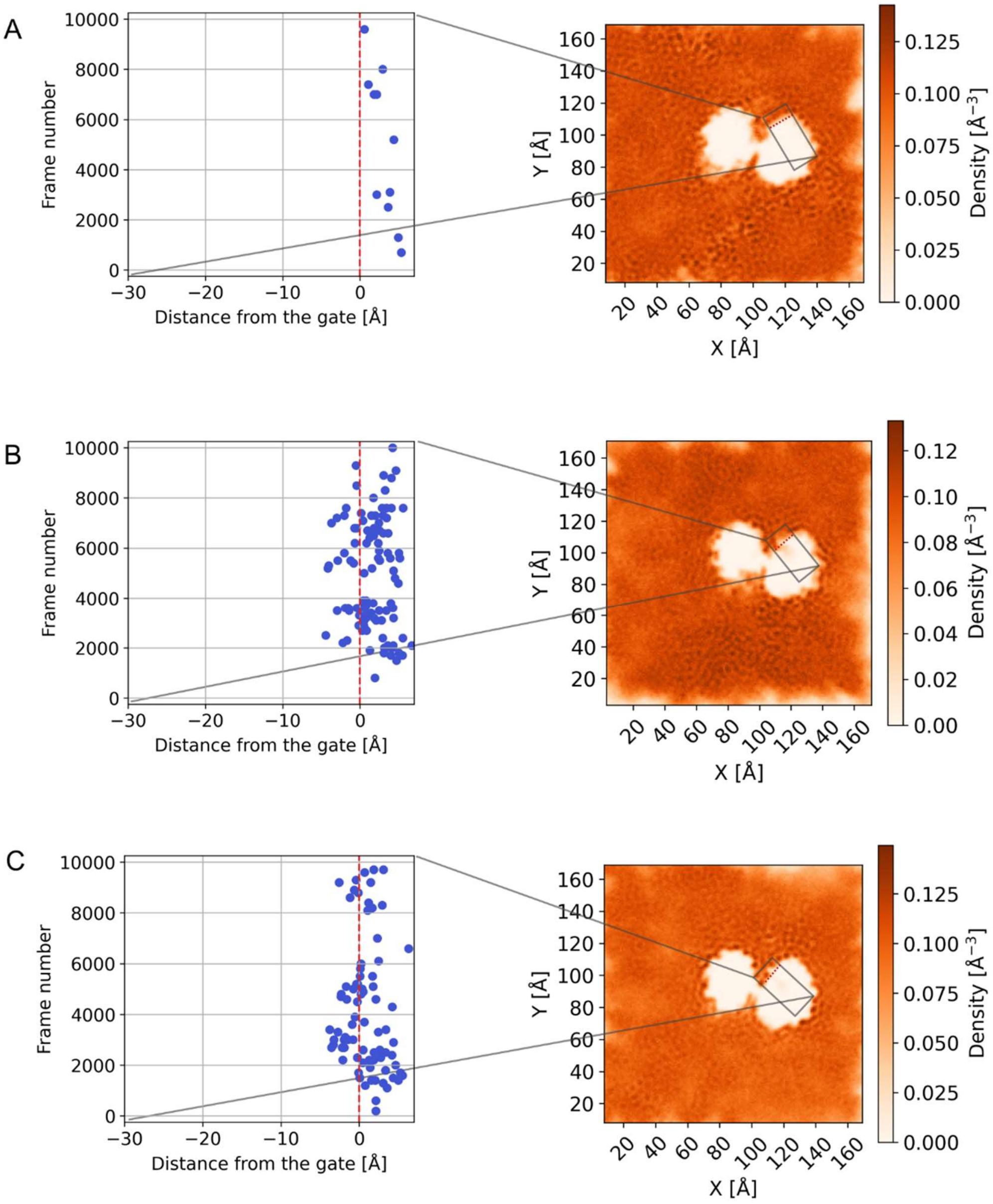

Figure S4. The GfcD C-terminal channel is fully hydrated in simulations. PyMOL render of water and protein from a representative simulation. Membrane lipids are hidden for visualization purposes.

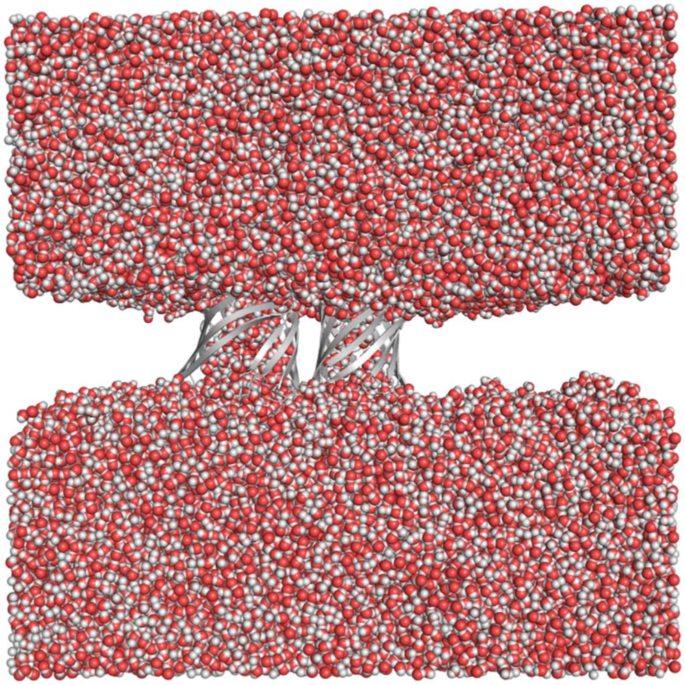

Figure S5. The lateral aperture of GfcD is stable in simulations of GfcD without C-terminal plug, with lipid A. (A) RMSD of the protein backbone. (B) RMSF of the protein backbone, with the secondary structure of GfcD highlighted in the background. (C) Three reference distances with which we measure the aperture (L437-W668, L433-W666, Y427-R663). (D) Time evolution of the three distances shown in Panel C.

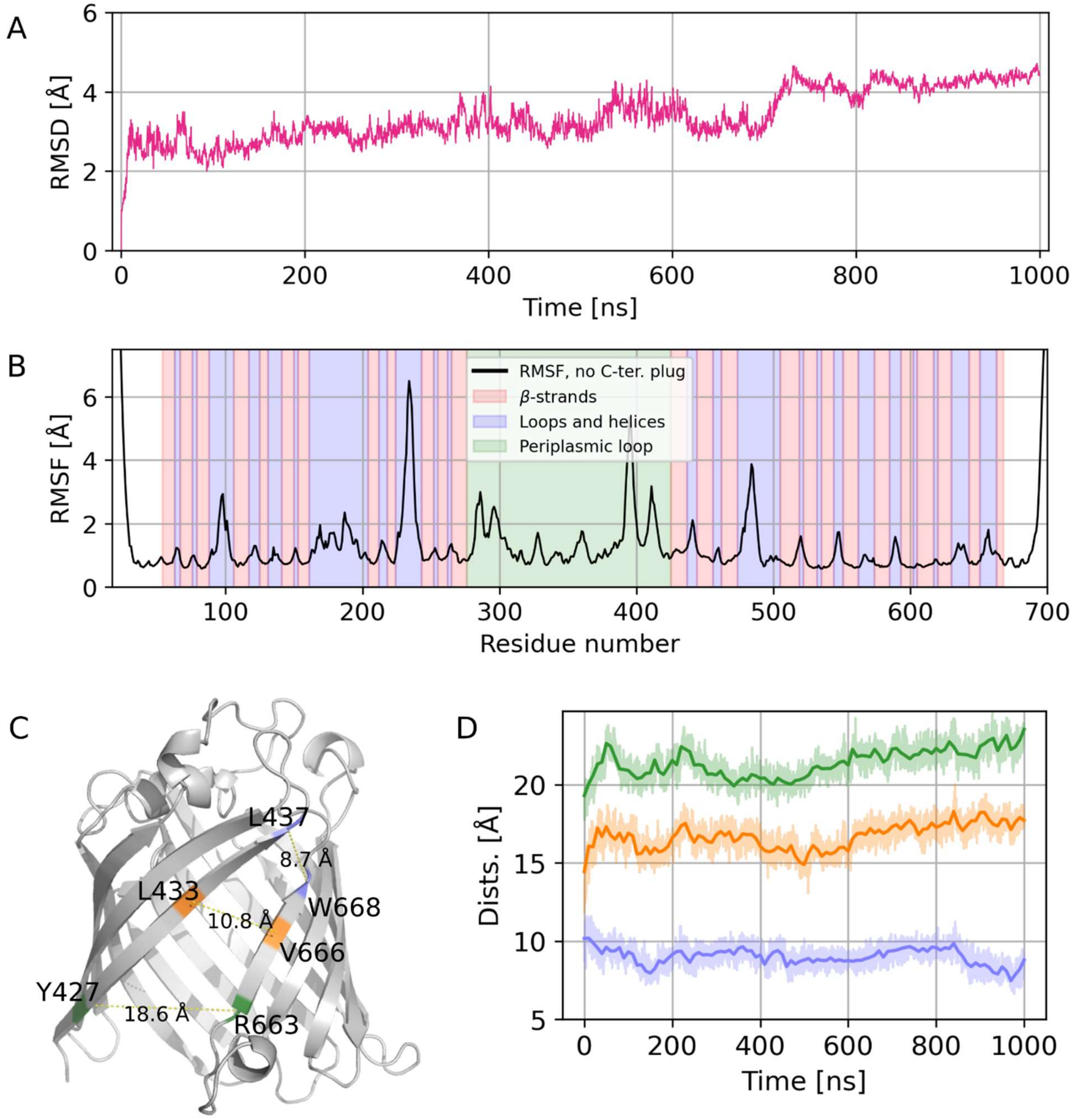

Figure S6. Analysis of the simulations of GfcD without C-terminal plug, with lipid A. (A) Tilting angle of lipid A (cf. Figure 4). (B) Insertion of lipid A aliphatic tails into the membrane (cf. Figure 5).

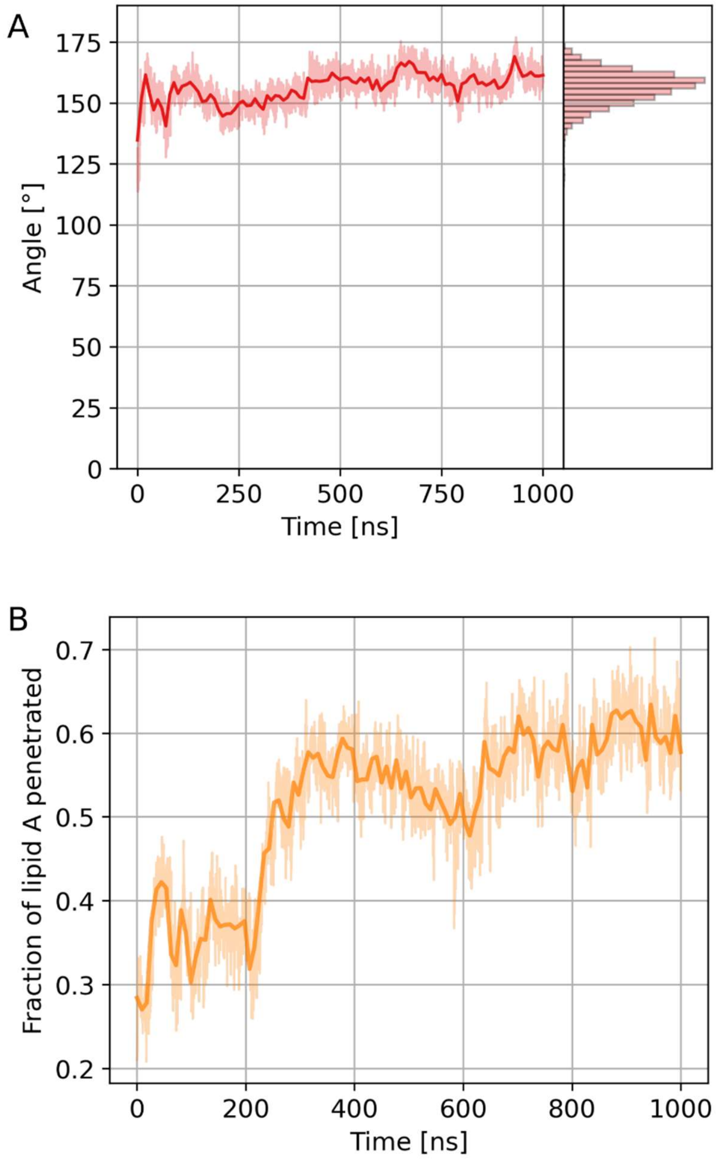

Supplement: Document S1. Figures S1–S6, Tables S1, and S2 [file mmc1.pdf]
